# To Our Dental Friends

**Published:** 1880-03

**Authors:** 


To Our Dental Friends.—We hope that they will not let our pages lack for original papers in their specialty. If they have not time to write long articles, we will be pleased to receive something short and terse. This number was filled before we could get sufficient matter in that department, and we trust that they will find it none the less acceptable.

